# The canonical Wnt pathway in osteoporosis: a scoping review of key compounds and proteins modulating Wnt-induced osteogenesis

**DOI:** 10.3389/fphar.2025.1669222

**Published:** 2025-11-07

**Authors:** Nyruz Elahmer, Norazlina Mohamed, Sok Kuan Wong, Nur Khadijah Muhamad Jamil, Isa Naina Mohamed, Sabarul Afian Mokhtar, Norliza Muhammad

**Affiliations:** 1 Department of Pharmacology, Faculty of Medicine, Universiti Kebangsaan Malaysia, Kuala Lumpur, Malaysia; 2 Department of Pharmacology, Pharmacy Faculty, Elmergib University, Al Khums, Libya; 3 Department of Pharmacology, Faculty of Medicine, Universiti Sultan Zainal Abidin, Kuala Terengganu, Malaysia; 4 Department of Orthopaedics and Traumatology, Faculty of Medicine, Universiti Kebangsaan Malaysia, Kuala Lumpur, Malaysia

**Keywords:** Wnt signaling, osteogenesis, osteoclastogenesis, bone formation, scoping review, β-catenin, osteoporosis

## Abstract

**Purpose:**

The canonical Wnt pathway—a key regulator of bone formation and remodeling—has emerged as a promising target for osteoporosis therapy. This scoping review aims to map the key compounds and proteins modulating Wnt-induced osteogenesis, providing a comprehensive overview of the current literature and identifying research gaps.

**Methods:**

A systematic search was conducted in Ovid and PubMed for studies published between June 2017 and August 2025. Two independent reviewers screened titles, abstracts, and full texts. Data were extracted and synthesised narratively.

**Results:**

Among 108 articles identified, 22 met the inclusion criteria. External compounds such as LG-HMF, cerium oxide nanoparticles, 6% Sr-MSNs, and platelet-rich plasma (PRP) were found to stimulate Wnt signaling, promoting osteogenesis and inhibiting osteoclastogenesis via diverse mechanisms. Molecular agents including β-sitosterol and fluoxetine also modulated the pathway, suggesting novel therapeutic opportunities. Internal regulators such as LINC01119, QKI, and PITX1 inhibited Wnt activity and were associated with bone loss, while GNAS, GCN5, and Ca(v)1.2 activated the pathway, enhancing bone health. The review highlights intricate crosstalk between canonical and Notch pathways and non-canonical Wnt pathways in bone remodeling. Clinical and epidemiological studies further confirmed the relevance of Wnt signaling by linking specific genetic and protein markers to bone mineral density and fracture risk.

**Conclusion:**

This scoping review highlights the dual role of Wnt pathway modulators—stimulators enhance bone formation, while inhibitors contribute to osteoporosis—emphasizing its potential in guiding targeted therapies and identifying genetic markers for personalized osteoporosis treatment.

## Introduction

Osteoporosis is a widespread condition affecting millions worldwide. It is characterized by reduced bone mass per unit volume and deterioration of bone microarchitecture, leading to increased fragility and susceptibility to fractures (Chin et al., 2022). A systematic review and meta-analysis encompassing 86 studies across five continents estimated the global prevalence of osteoporosis at 18.3%, underscoring its significant public health burden (Salari et al., 2021). The condition disproportionately affects women, particularly postmenopausal individuals, due to the decline in estrogen levels—a hormone essential for maintaining bone health (Chan et al., 2022). However, men are not exempt from osteoporosis, with studies revealing gender-specific differences in knowledge, motivation for physical activity, and barriers to preventive measures (Chan et al., 2019). A cross-sectional study reported an overall prevalence of 15.3%, with higher rates observed in women (18.9%) compared to men (11.5%). These findings highlight gender disparities in osteoporosis prevalence and underscore the need for sex-specific public health interventions (Subramaniam et al., 2019).

The pathophysiology of osteoporosis is complex and multifactorial, involving more than just an imbalance between bone resorption by osteoclasts and bone formation by osteoblasts. In addition to dysregulated bone remodeling—marked by increased resorption or decreased formation—various intrinsic and extrinsic factors contribute to disease progression. These include mechanical loading, hormonal influences, metabolic regulators such as Glucagon-Like Peptide-1 (GLP-1), post-translational modifications, and microRNAs, all of which interact with core signaling pathways like RANK/RANKL/OPG, parathyroid hormone (PTH), cytokine networks (Peake et al., 2021), and notably, the Wingless-int (Wnt)/β-catenin signaling pathway (Zhao et al., 2018). A comprehensive understanding of these interconnected mechanisms is essential for advancing targeted and effective therapeutic strategies.

The canonical Wnt pathway is one of the most well-characterized signaling cascades in skeletal biology, playing a central role in bone formation and remodeling. This pathway is initiated by the binding of Wnt ligands to Frizzled receptors and their co-receptors, Low-Density Lipoprotein Receptor-Related Protein 5/6 (LRP5/6), on the cell surface (Janssen et al., 2021). This interaction inhibits glycogen synthase kinase-3β (GSK-3β), preventing the ubiquitination and subsequent degradation of β-catenin (Feehan et al., 2019). Stabilized β-catenin translocates into the nucleus, where it interacts with T-cell factor/lymphoid enhancer-binding factor (TCF/LEF) transcription factors to regulate the expression of genes critical for osteoblast differentiation and function (Liu et al., 2016). Wnt signaling is pivotal in promoting osteoblastogenesis while simultaneously inhibiting osteoclastogenesis. Activation of β-catenin enhances the differentiation of mesenchymal stem cells (MSCs) into osteoblasts while suppressing their differentiation into adipocytes or chondrocytes. Additionally, β-catenin increases the osteoprotegerin (OPG) to receptor activator of nuclear factor kappa-Β ligand (RANKL) ratio, thereby reducing osteoclast activity and bone resorption (Liu et al., 2016). Mutations in components of this pathway profoundly impact bone mass; for example, loss-of-function mutations in LRP5 result in low bone mass syndromes, while gain-of-function mutations lead to high bone mass phenotypes (Garcia de Herreros and Duñach, 2019). These findings highlight the critical role of Wnt signaling in maintaining skeletal homeostasis. In this review, we delve into the role of the canonical Wnt pathway in osteoporosis, emphasizing key compounds, proteins, and genes that modulate Wnt-induced osteogenesis. By examining both natural and pharmacological modulators of this pathway, we aim to provide insights into how these factors influence bone metabolism and their potential as therapeutic targets. Furthermore, this review underscores the promise of Wnt-targeted strategies as a novel approach for addressing osteoporosis at its root cause—by restoring balance to bone remodeling processes. By advancing our understanding of this pathway, we hope to contribute to the development of innovative treatments that could transform osteoporosis management and improve patient outcomes.

## Materials and methods

### Search strategy and selection criteria

This scoping review was designed and reported in accordance with the Preferred Reporting Items for Systematic Reviews and Meta-Analyses extension for Scoping Reviews (PRISMA-ScR) guidelines (Tricco et al., 2018) and followed the methodological framework proposed by Arksey and O'Malley (2005). A comprehensive literature search was conducted across Ovid and PubMed databases, covering health science journals published between 5 June 2017, and 28 August 2025. The search strategy combined two sets of keywords: (1) the Wnt pathway AND (2) osteo*, with the restriction that both terms appear in the title. Only studies published in English were included. Eligible studies met the following inclusion criteria: (1) they investigated the role of the Wnt pathway in osteogenesis and/or osteoclastogenesis, and (2) they examined how either external interventions or endogenous proteins and genes modulate the Wnt pathway in bone cells. Review articles, letters, editorials, news articles, and case studies were excluded from the analysis.

### Data extraction and management

Two independent reviewers screened titles, abstracts, and full texts for eligibility. Articles underwent a three-step screening process before inclusion. In the first step, publications that did not meet the inclusion criteria based on their titles were excluded. In the second step, abstracts of the remaining articles were reviewed, and those that failed to meet the criteria were removed. Finally, full-text articles were obtained and thoroughly examined to exclude any that did not align with the inclusion requirements. After removal of duplicates, the remaining articles underwent a secondary screening by the two reviewers. Full articles required approval from both reviewers before proceeding to the data extraction stage. Any disagreements were resolved through discussion. Data extraction was carried out independently using a standardised data collection form. The extracted information included: (1) the study type, (2) the type of treatment examined, (3) a brief description of the study sample or population, (4) an overview of the procedures used, and (5) a summary of the study’s findings.

## Results

### Search results

**FIGURE 1:**
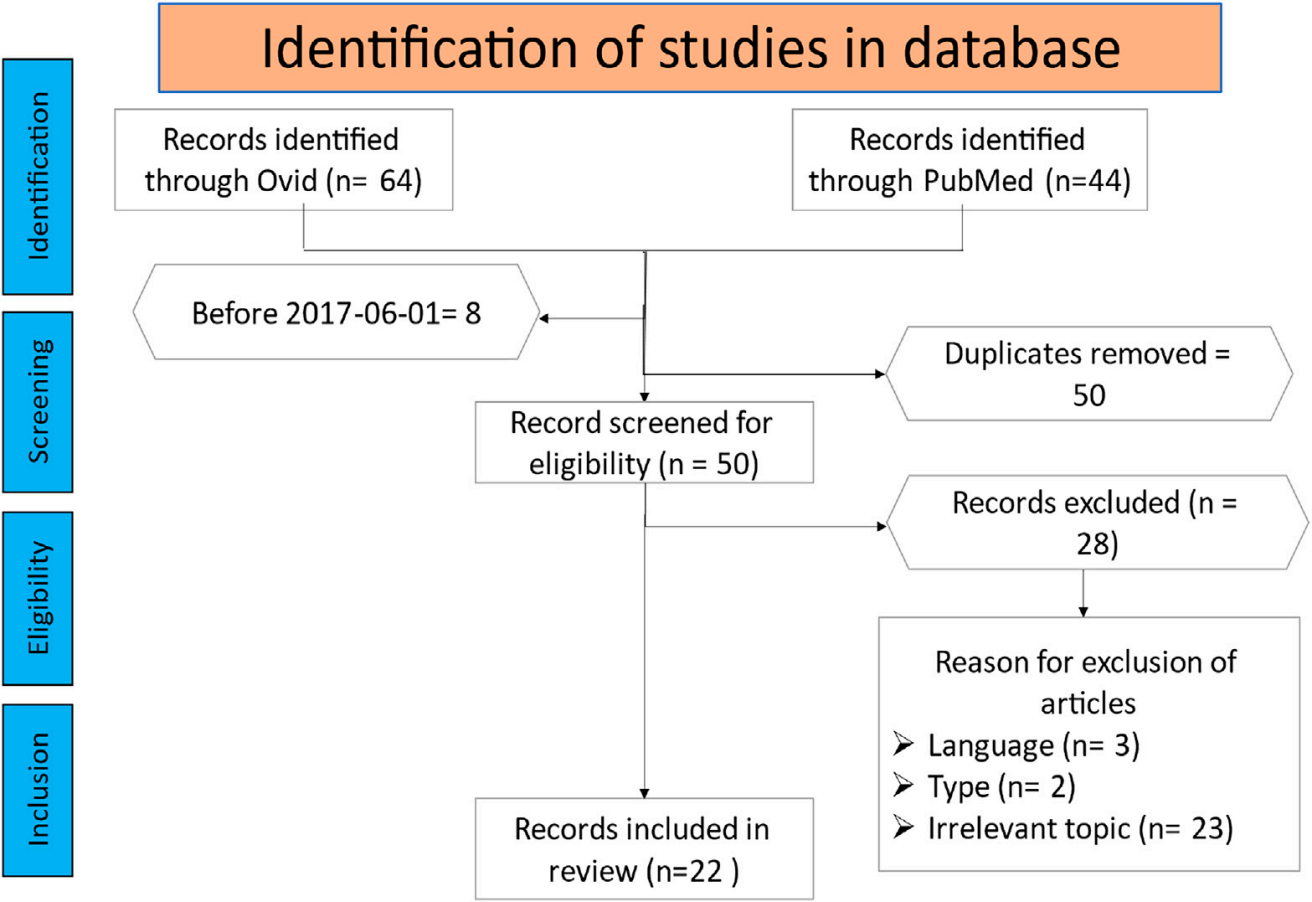
The flowchart illustrates the article selection procedure for this scoping review.

The literature search identified 108 potential publications. All articles before fifth of June 2017 were excluded. The two reviewers independently evaluated the titles and abstracts to determine their eligibility for inclusion in the scoping review. After the screening, 22 publications were deemed appropriate for further analysis and data extraction. The remaining papers were excluded for various reasons, such as lack of relevance to the role of the Wnt pathway in osteoporosis, language, or article type. Figure 1 provides an overview of the article selection process and the reasons for exclusion.

### Study characteristic

Table 1 provides a summary of the main characteristics of the included studies published between 2017 and 2025. These include nine *in vitro* studies, two human studies, one epidemiological study, and ten studies combining animal and *in vitro* experiments. With the exception of two studies using zebrafish larvae, mice were the primary animal model employed. The mice underwent various procedures to simulate different forms of osteoporosis, including genetic modification, ovariectomy, surgical interventions, administration of dexamethasone sodium phosphate, or treatment with Dkk-1. The *in vitro* experiments utilized a variety of cell lines, such as MLO-Y4, RAW 264.7, MC3T3-E1, 293T, IDG-SW3, UMR-106, ST2, C2C12, Human BMSCs, Rat BMSCs, Mouse BMMSCs, SCAPs, DPSCs, HUVECs, and BMMs. In the human study, comparisons were made between osteoporotic patients and healthy controls. All studies employed experimental designs that compared treatment groups, exposed to various compounds such as cerium oxide nanoparticles (Ceo2Nps), 6% strontium doped mesoporous silica nanoparticles (6% Sr-MSNs), and platelet-rich plasma (PRP) with control groups to examine the effects of the Wnt signaling pathway on bone cell development.

**TABLE 1:** The evidence table presents a summary of included studies, including key characteristics and findings.

Study title	Study design	Findings
Cell culture studies		
Magneto-mechanical stimulation modulates osteocyte fate via the ECM-integrin-CSK axis and wnt pathway (Zhang et al., 2023)	Cell: Murine Osteocyte-like Cell Line MLO-Y4 Pre-osteoclast RAW 264.7 cells Mode of disease induction: NON Treatment: 12 T large gradient high-static magnetic fields (12T LG-HMFs) in various directions relative to gravity for 24, 48, and 72 hours Control: Negative: no treatment (did not receive any magnetic force stimulation) Positive: no	12 T LG-HMF upward treatment: ↓Osteocyte proliferation ↑Cell apoptosis ↓Wnt1, b-catenin, and LRP6 ↑SOST ↓NO and OPG ↑Osteoclastogenesis 12 T LG-HMF downward treatment: ↑Osteocyte growth ↓Cell apoptosis ↑NO ↓SOST ↓Osteoclastogenesis
Cerium Oxide Nanoparticles Promote Osteoplastic Precursor Differentiation by Activating the Wnt Pathway (Luo et al., 2023)	Cell: MC3T3-E1 mouse precursor osteoblasts cells Mode of inhibition induction: Inhibition of the Wnt pathway using KYA1797K reversed the stimulative effect of CeO2 NPs Treatment: 1 µg/mL Cerium Oxide Nanoparticles CeO2 NPs for 48 h Control: • Negative: no treatment • Inhibitor negative: KYA1797K • Positive: no	↑Matrix mineralization ↑Ca2+ accumulation ↑Osteogenic genes (Runx2, Col Iα1, OCN) ↑Osteogenic differentiation ↑Fam53B gene expression ↓Osteogenic differentiation AFTER KYA1797K
LINC01119 negatively regulates osteogenic differentiation of mesenchymal stem cells via the Wnt pathway by targeting FZD4 (Gao et al., 2022)	Cell: Human BMSCs (hBMSC) & 293 T cell line Database: GSE80614 dataset GEO website databas, Jaspar database. UCSC and the Lisa database Analytical tool: Bioconductor packages (impute and limma) & R software, DAVID Bioinformatics Tool Data type: Genomic data (66 samples) Mode of inhibition and activation induction: overexpressing and knocking down of LINC01119 and FZD4 in mesenchymal stem cells (MSCs) Treatment: specific short hairpin RNAs (shRNAs) AND lentivirus infection Control: • Negative: no treatment • Positive: no	LINC01119 Knockdown: ↑Calcium deposition ↑Osteogenic differentiation ↑RUNX2, OSX, OCN, & ALP activity ↑LRP5 and beta-catenin LINC01119 overexpression: ↓Mineralized nodule ↓RUNX2, OSX, OCN, & ALP activity FZD4 overexpression: ↑Osteoblast proliferation ↑Calcium deposition ↑RUNX2, OSX, OCN, & ALP activity FZD4 knockdown & LINC01119 knockdown: ↓Calcium deposition ↓RUNX2, OSX, OCN, & ALP activity EBF3 knockdown: ↓Binding of EBF3 and LINC01119
Noncanonical Wnt5a Signaling Suppresses Hippo/TAZ-Mediated Osteogenesis Partly Through the Canonical Wnt Pathway in SCAPs (Fu et al., 2022)	Cell: Stem cells from the apical papilla (SCAPs) Mode of activation induction: Osteogenic inducing medium (OIM) Treatment: 100 ng/mL Wnt5a for 7 days and lentiviruses & HitransG P for 9 h Control: • Negative: no treatment • Positive: no	Wnt5a treated group: ↓Bone-related factors (ALP, RUNX2, TAZ) ↓Mineralization nodule formation ↓Osteogenesis Under an OIM, Wnt5a treated group: ↓TAZ expression Overexpress TAZ group: ↑ALP activity ↑Mineralized nodules formation ↑Bone-related factors ↑Osteogenesis Overexpress TAZ group plus Wnt5a: ↓ALP activity ↓Mineralized nodules ↓Bone-related factors Overexpress β-catenin group: ↑TAZ Overexpress β-catenin group plus Wnt5a: ↓TAZ ↓ALP activity ↓Mineralized nodules ↓Bone-related factors
Downregulation of GNAS inhibits osteogenesis of bone marrow mesenchymal stem cells and promotes osteoporosis through the Wnt pathway (Zheng et al., 2020)	Cell: BMSCs Mode of disease induction: Using osteoporosis (OP) tissue Treatment: sh-GNAS to knockdown GNAS & LV-GNAS to overexpress GNAS for 6–8 h Control: • Negative: no treatment • Positive: no	OP tissues: ↓GNAS levels Osteogenesis induction media: ↑GNAS levels Knockdown GNAS group: ↓Osteogenesis-related factors (ALP & RUNX2) ↓ALP activity ↓Mineralization capability ↓Wnt3a and β-catenin Overexpress GNAS group: ↑ALP activity ↑Runx2 ↑Mineralization capability ↑Wnt3a and β-catenin Overexpress Wnt3a plus knockdown GNAS group: ↑Osteogenesis-related factors (ALP & RUNX2) ↑ALP activity ↑Mineralization capability ↑Wnt3a and β-catenin
TNF-α was involved in calcium hydroxide-promoted osteogenic differentiation of human DPSCs through NF-κB/p38MAPK/Wnt pathway (Cui et al., 2017)	Cell: dental pulp stem cells (DPSCs) Mode of inhibition induction: 10 mmol/L NF-κB inhibitor (PDTC), an Wnt inhibitor (DKK1), or a p38 inhibitor SB203580) for 1 h Treatment: 10 mg/mL calcium hydroxide for 24 h or 14 days Control: • Negative: treated with a DMSO • Positive: no	Ca (OH)_2_: ↑Cell viability ↑Cell migration ↑ALP expression ↑Mineralization ↑p-IKBA ↑p-p38 ↓p-GSK3β ↑^+^ DSPP, DMP1, OPN and ALP Ca (OH)_2_ + PDTC, or DKK1, or SB: ↓Cell viability ↓Cell migration ↓ALP expression ↓Mineralization Ca (OH)2 + PDTC: ↑DSPP, DMP1, OPN and ALP Ca (OH)2 + DKK1: ↑OPN and ALP Ca (OH)2 + SB: ↑ALP Ca (OH)2 + siTNF-α: ↓p-IKBA ↓p-p38 ↑p-GSK3β
Cytoprotective Effect of Taurine against Hydrogen Peroxide-Induced Oxidative Stress in UMR-106 Cells through the Wnt/β-Catenin Signaling Pathway (Lou et al., 2018)	Cell: Rat osteogenic sarcoma line UMR-106 Mode of disease induction: H_2_O_2_ (100 μM) for 24 h to induce stress Mode of Inhibition induction: Treatment of DKK1 (100 ng/mL) for 1 h before taurine Treatment: taurine (0, 10, 50, 100, and 200 mM) for 3 h before H_2_O_2_-induced stress Control: • Negative: No treatment. • Positive: Treated with H_2_O_2_ (100 μM)	↑ Osteoblast cell viability ↑ ALP activity ↑ β-catenin ↑ ERK ↑ HO-1, NQO1, GCLC ↑ Nrf2
The regulatory roles of Notch in osteocyte differentiation via the crosstalk with canonical Wnt pathways during the transition of osteoblasts to osteocytes (Shao et al., 2018)	Cell: Rat BMSCs, IDG-SW3 cells, MC3T3-E1 and 293T cell lines Mode of Activation induction: Using Notch1 extracellular antibody-coated cell culture plate & transfection of a Hairy/Enhancer of Split 1 (Hes1) overexpression vector Treatment: 5 mM LiCl to activate Wnt signaling and 50 μM DAPT to inhibit Notch signaling pathway Control: • Negative: treated with a DMSO. • Positive: no	Notch stimulated group: ↓late-stage osteoblast proliferation ↓p-Akt Notch stimulated group + LiCl: ↓β-catenin Notch stimulated group + DAPT: ↑β-catenin ↓GSK-3β ↑p-Akt
(R)‑dehydroxyabscisic alcohol β‑D‑apiofuranosyl‑(1″→6′) β‑ D‑glucopyranoside enhances the osteoblastic differentiation of ST2 cells via the BMP/WNT pathways (Liu et al., 2019)	Cell: ST2 bone marrow stromal cell line Mode of inhibition induction: Noggin (10 µg/ml) or Dkk-1 (0.5 µg/mL) for 1 h to block BMP pathway and Wnt pathway, respectively Treatment: 0.1, 0.5 or 2.5 µM DAG for different time periods according to the assays 5, 7, 10, and 14 days. Control: • Negative: treated with DMSO • Positive: no	DAG treatment group: ↑ALP activity ↑Calcium deposits ↑Alp, Ocn, & Opn ↑Mineralized nodule ↑Bmp2 & Bmp4 ↑p-Smad1, 5 & 8 ↑Wnt1 & 3 ↑β-catenin ↑Runx2 Noggin + DAG treatment group: ↓ALP ↓Mineralization ↓p-Smad1, 5 & 8 ↓Runx2 Dkk‑1 + DAG treatment group: ↓ALP ↓β-catenin ↓Runx2
Combined animal and in vitro studies:		
Strontium doped mesoporous silica nanoparticles accelerate osteogenesis and angiogenesis in distraction osteogenesis by activation of Wnt pathway (Liu et al., 2022)	Cell: Human BMSCs and HUVECs Animals: 54 adult male Sprague–Dawley (SD) rats (350–450 g) Mode of disease induction: BMSCs were isolated and cultivated to provide a source of cells that could interact with HUVECs to mimic the bone healing environment in in vitro A transverse midshaft right tibial osteotomy was performed and installed mono-lateral external fixator in in vivo Treatment: 250 μg/mL 6% in in vitro 6% Sr-MSNs (100 μg in 100 μL of PBS) into the distraction gaps once a week after distraction in in vivo Control: • Negative: treat with PBS • Positive: treat with 250 μg/mL MSNs in in vitro and MSNs (100 μg in 100 μL of PBS) in in vivo	6% Sr-MSNs treated BMSCs group: ↑ALP ↑ALP activity ↑Calcium deposition ↑Mineralization ↑Osteogenesis-related genes (Runx2, BMP2, Col-1, ALP, OPN, & OCN) ↑Wnt/catenin pathway genes (c-myc, pGsk-3β & β-catenin) ↓Gsk-3β ↑OPG ↓RANKL 6% Sr-MSNs treated SD rats’ group: ↑Callus formation. ↑BMD ↔BV/TV ↑Collagen deposition ↑OCN, Col-1, & OPG Conditioned medium from 6% Sr-treated BMSCs treated HUVECs group: ↑vascular endothelial growth factor (VEGF) ↑Proliferation ↑Tube network ↑Neoangiogenic vessels ↑CD 31, a-SMA, & VEGF
RNA-binding Protein QKI Inhibits Osteogenic Differentiation Via Suppressing Wnt Pathway (Yan et al., 2023)	Cell: Primary BMSCs Animals: All were Male C57BL/6J mice, 12 weeks old *Prx1-Cre +; Rosa ^QKI/ +^ mice, Prx1-Cre+; Rosa ^QKI^ mice (for overexpression) *Prx1–Cre–; Rosa ^QKI/+^ mice (control) *Prx1-Cre +; QKI ^fl/fl^ mice (for knockdown) * Prx1-Cre-; QKI ^fl/fl^ littermate control mice Mode of disease induction: Glucocorticoid-induced osteoporosis by 50 mg/kg of dexamethasone sodium phosphate injection, daily for 4 weeks Mode of inhibition and activation induction: treat with IWR1 to inhibit Wnt pathway and SKL2001to activate Wnt pathway, Treatment: genetically modification treatment (QKI knockout or overexpression). Control: • Negative: no treatment • Positive: no	QKI knockout: ↑Osteogenic differentiation ↑Bone formation ↑BMD, BV/TV, Tb.Th, & Tb.N QKI knockout + IWR1: ↓Osteogenic differentiation ↓ALP ↓Runx2 QKI overexpression: ↓Osteogenic differentiation ↓Bone formation ↓ALP activity ↓Runx2 ↓Wnt pathway-related genes (Fzd7, Dvl3, & β-catenin) QKI overexpression + SKL2001: ↑Osteogenic differentiation ↑ALP ↑Runx2
Epigenetic inhibition of Wnt pathway suppresses osteogenic differentiation of BMSCs during osteoporosis (Jing et al., 2018)	Cells: Murine bone marrow mesenchymal stem cells (BMSCs) were flushed from long bones Animals: 80 eight-week female C57BL/6 mice, 22 six-week female immunocompromised nude mice (BALB/c) & 22 eight-week female C3H mice Mode of disease induction: surgical removal of ovaries in mice to mimic postmenopausal osteoporosis Treatment: 20 μL Intra-femoral injection of lentivirus vectors, Wnt3a to promote osteogenic differentiation, while repressing Wnt signaling by DKK1. Control: • Negative: no treatment • Positive: no	OVX mice: ↓Bone formation ↓Osteogenic differentiation ↓Active beta-catenin ↓Axin2 ↓Wnt1, Wnt6, Wnt10a, & Wnt10b ↓ H3K9ac ↓GCN5 Osteogenic induced BMSCs+ overexpression of Gcn5: ↑Osteogenic differentiation ↑OCN ↑β-catenin ↑H3K9ac ↑Wnt1, Wnt6, Wnt10a, and Wnt10b Osteogenic induced BMSCs+ knockdown of Gcn5: ↓Osteogenic differentiation ↓β-catenin ↓H3K9ac
Bone-Specific Overexpression of PITX1 Induces Senile Osteoporosis in Mice Through Deficient Self-Renewal of Mesenchymal Progenitors and Wnt Pathway Inhibition (Karam et al., 2019)	Cells: mesenchymal stem cells (MSCs) Animals: Ten transgenic mCol1α1-Pitx1 mic (females = 5; males=5) and ten wild type mice (females=5; males=5) of 12 weeks of age Mode of activation induction: 2.3 kb fragment of the mColIα1 promoter to overexpress Pitx1 coding sequence Treatment: 0.01 M of lithium chloride. Control: • Negative: no treatment or treated with NaCl [0.01M] • Positive: no	Pitx1 overexpression mice: ↓Bone formation ↓Bone mass density ↓Biomechanical bone strength ↓Bone length ↓Bone mineral content ↓Bone microarchitecture ↓Runx2, Osx, Alp, & OCN ↓Mesenchymal precursors ↓RANKL ↑OPG ↑Osteoclast number ↑Sost & Dkk1 ↑Gsk3β ↑phospho-β-catenin ↓Axin 2 Silencing Pitx1 expression: ↑Osteoblast differentiation ↑Mineralization ↑Runx2, Osx, Alp & Spp1 mCol1α1-Pitx1 mice + LiCl: ↓Bone loss
Ca(v) 1.2 regulates osteogenesis of bone marrow‐derived mesenchymal stem cells via canonical Wnt pathway in age‐related osteoporosis (Fei et al., 2019)	Cells: Primary BMMSCs. Animals: 3‐month‐old male wild type and Zmpste24−/− mice. Mode of disease induction: Genetically modification of mice, leading lacking the Zmpste24 gene to mimic age related osteoporosis. Treatment: 10^−7^ M Bay K8644 treatment (the potent L‐type Ca2+ channel agonist) for BMMSCs for 7 & 14 days & intraperitoneal injection of 1 mg/kg Bay K8644 for mice treatment for two months. Control: • Negative: treat with DMSO • Positive: no	Zmpste24−/− mice: ↓Mineralization ↓Bone mineral density (BMD) ↓Bone volume/total volume (BV/TV) ↓Trabecular number (Tb.N) ↓Bone formations ↓Ca _V_1.2 Zmpste24−/− BMMSCs: ↓ALP, Runx2, & OCN ↓Mineralized nodules ↓VGCCs genes (Ca _V_1.2 & Ca _V_ 2.2) ↓p‐GSK3β ↓Active‐β‐catenin ↓Wnt target genes of cyclin D1 & c‐myc BMMSCs from old individuals: ↓Ca _V_1.2 Cav1.2 siRNA/wild‐type BMMSCs: ↓Osteogenic related genes (ALP, Runx2, & OCN) ↓P‐GSK3β ↓Active‐β‐catenin ↓Wnt target genes of cyclin D1 & c‐myc Cav1.2 overexpression vector/Zmpste24−/− BMMSCs: ↑Osteogenic differentiation ↑Mineralized nodules ↑P‐GSK3β ↑Active‐β‐catenin ↑Wnt target genes of cyclin D1 & c‐myc Zmpste24−/− BMMSCs with LiCl: ↑Calcified nodule Zmpste24−/− BMMSCs with Bay K8644: ↑p‐GSK3β ↑Active‐β‐catenin ↑Wnt target genes of cyclin D1 & c‐myc ↑Mineral node formation Zmpste24−/− mice with Bay K8644: ↑BMD, BV/TV, & Tb.N ↑Bone formation
Platelet-rich plasma inhibits RANKL-induced osteoclast differentiation through activation of Wnt pathway during bone remodeling (Wang et al., 2018)	Cells: Bone marrow cells and hematopoietic cells BMMs. Animals: 100, 3-week-old female BALB/c mice. Mode of disease induction: The lower incisors were trimmed by 1 mm at the incisal third every 3 days until the mice were sacrificed. Treatment: PRP is injected once a week for 7, 14 and 28 days in in vivo. 1% PRP for 3, 4, and 12 days in in vitro. Control: • Negative: normal saline injection • Positive: no	PRP group: ↑BV/TV ↑Tb.N ↓Tb.Sp ↔Tb.Th ↓Mature osteoclasts ↑Osteoblast formation ↑Mineralization nodules ↓ ↓Resorptive activity genes (Ctsk, CAR2 & MMP9) ↑β-catenin & cyclin D1 ↓Dkk1
Irisin reshapes bone metabolic homeostasis to delay age-related osteoporosis by regulating the multipotent differentiation of BMSCs via Wnt pathway (Xing et al., 2025)	Cells: Bone marrow from the femur and tibia Animals: Male C57BL/6 mice (2 and 15 months old) Mode of disease induction: mice at two different ages Mode of inhibition induction: iwp2 (a potent Wnt pathway inhibitor) Treatment: 25 and 100 mg/kg of 5 ng/mLirisin (IV) once a week for 4 weeks Control: • Negative: Young-Vehicle group. • Positive: Aged-Vehicle group.	↑ Calcium deposition at day 14 ↑ Runx2 and ALP ↑ β-catenin protein and gene ↑ BMD, BV/TV, BS/TV, Tb.Th, and Tb.N. ↓ Tb. Sp ↑ Osteoid formation ↑ Bone mass recovery ↑ OCN gene
Protosappanin B activates the Wnt pathway to protect against glucocorticoid-induced osteoblast inhibition and enhance bone formation (Fan et al., 2025)	Cells: MC3T3-E1 pre-osteoblast cells Animals: Wild-type zebrafish (AB strain) Mode of disease induction: Zebrafish larvae was exposed to 25 mg/L of prednisolone to induce glucocorticoid-induced osteoporosis. 10 μM Dexamethasone was used to induce osteoporosis in MC3T3-E1cells Mode of inhibition induction: XAV939 (a Wnt pathway inhibitor) Treatment: 5, 10, and 20 μM Protosappanin B Control: • Negative: E3 medium alone • Positive: No	↑ Osteoblast proliferation ↓Osteoblast apoptosis rates ↑ Calcium deposition ↑ ALP ↑ Runx2, Osterix, and Osteocalcin genes and proteins ↑ β-catenin protein ↑ Bone formation
Schisandrin A induces osteoblast differentiation to treat glucocorticoid-induced osteoporosis through activating Wnt pathway (Ai et al., 2025)	Cells: MC3T3-E1 mouse osteoblast cell line Animals: Sixty 2-month-old SPF-grade female Balb/c mice Mode of disease induction: intraperitoneal injection of 1 mg/kg dexamethasone, daily for 4 weeks in in vivo. 10 μM dexamethasone in in vitro Mode of inhibition induction: 10 μM Box5 (a Wnt5a inhibitor) Treatment: 25, 50, 100 mg/kg Schisandrin A for 4 weeks in in vivo. 50 μM Schisandrin A in in vitro Control: • Negative: 0.9 % saline • Positive: 0.9 mg/kg alendronate sodium	↑ Tb.N, Tb.Th, BMD, and BV/TV ↑ Bone mass ↑ Serum Ca and P levels ↑ Bone collagen fiber content ↑ Runx2 and Osterix ↑ Serum ALP levels ↑ Fzd4, Tcf7l1, Wnt5a, and β-catenin protein ↓ Apcdd1, Cxxc4, Nkd2, Sfrp2, and Sfrp5 ↑ Osteoblast cell viability ↑ Luciferase activity
Acceleration of osteoblast differentiation by a novel osteogenic compound, DMP-PYT, through activation of both the BMP and Wnt pathways (Bae et al., 2017)	Cell: Mouse cell lines C2C12 and MC3T3-E1 subclone 4 Animals: zebrafish larvae Mode of inhibition induction: treat with 100nM of LDN-193189, the BMP inhibitor Treatment: 2.5 µM, 5 µM & 10 µM DMP-PYT for 4 days in in vivo and 5 µM &10 µM DMP-PYT for 30 min, 4 h, 24 h & 48h in in vitro Control: • Negative: treated with a vehicle. • Positive: Treat with BMP2 (25 ng/mL) or Wnt3a (50 ng/mL).	MP-PYT: ↑ALP activity MP-PYT + BMP2: ↑BMP2-induced osteoblast differentiation ↑BMP2-induced β-catenin ↑pSMAD1/5/8 BMP2: ↑pSMAD1/5/8 ↑β-catenin ↑Osteoblast-specific markers ALP, osterix, osteocalcin, & RUNX2 ↑BMP2, BMP4, BMP6, & BMP7 ↑pSMAD1/5/8 DMP-PYT: ↑^+^ ALP activity ↑^+^Osteoblast differentiation ↔BMPR1β & BMPRII ↔BMP3 & Activin ↑β-catenin-activated TCF/LEF DMP-PYT + BMP2: ↑^+^BMP2-induced osteoblast differentiation ↑^+^Osteoblast-specific markers ALP, osterix, osteocalcin, & RUNX2. ↑^+^BMP2, BMP4, BMP6, & BMP7 ↑^+^Wnt3a ↑^+^pSMAD1/5/8 ↑β-catenin-activated TCF/LEF DMP-PYT+ LDN-193189: ↓pSMAD1/5/8 ↓Osteoblast differentiation ↓Osteogenic markers DMP-PYT + Wnt3a: ↑β-catenin-activated TCF/LEF DMP-PYT treated Zebrafish larvae: ↑Skeletal development
Epidemiological study		
Investigating the impact of Wnt pathway‑related genes on biomarker and diagnostic model development for osteoporosis in postmenopausal females (Lai et al., 2024)	Database: microarray datasets (GSE56814, GSE56815, GSE2208) from the GEO website database & Reactome pathway database & DGIdb database for postmenopausal unrelated Caucasian females Analytical tool: R package, “DALEX” package, “caret” package, “GSVA” package Data type: Genomic data (329 Wnt pathway-related genes) Diagnostic models: Support vector machine (SVM), random forest (RF), generalized linear model (GLM), and XGB model.	low BMD group: ↑Wnt pathway-related genes (RAC1, KLHL12, GSK3B, ITPR2, & PPP2R5B) ↓Other 7 Wnt pathway-related genes
Clinical Study		
The Wnt pathway regulator expression levels and their relationship to bone metabolism in thoracolumbar osteoporotic vertebral compression fracture patients (Ma et al., 2021)	Population: 40 healthy controls (group A), 33 osteoporotic patients (group B), and 47 thoracolumbar OVCF patients Sample Size: 120 patients Study Design: Cohort Treatment: no Control: • Negative: healthy patients • Positive: no	OVCF group: ↓Bone density ↓levels of IL-10 & TGF-β1 ↑levels of leptin, IL-2, TNF-α, & IL-6 ↓β-catenin ↑DKK-1 ↑Bone resorption markers (MMP-2, MMP-9, RANKL, & β-CTX) ↓Bone formation marker levels (PINP, OPG, BALP, & BGP)
The genetic polymorphisms of key genes in WNT pathway (LRP5 and AXIN1) was associated with osteoporosis susceptibility in Chinese Han population (Cui et al., 2022)	Population: 1198 Chinese Han people who were recruited from the Second Affiliated Hospital of Xi'an Jiaotong University Sample Size: 599 osteoporosis patients and 599 healthy individuals Study Design: case-control study Treatment: no Control: • Negative: healthy patients. • Positive: no	LRP5 rs11228240: ↓Osteoporosis by more than half AXINI rs9921222: ↑Osteoporosis by more than twice AXINI rs2301522: ↑Osteoporosis

### Exogenous modulators and interventions targeting wnt signaling

Exogenous interventions demonstrated diverse regulatory effects on bone cells, with most studies focusing on osteoblast differentiation and mineralization, and a smaller subset addressing osteoclastogenesis and bone remodeling balance. Both *in vitro* models (MC3T3-E1, hFOB1.19, DPSCs, C2C12, ST2, MLO-Y4, RAW264.7, BMMs) and *in vivo* models (zebrafish, rats, mice) were utilized. Positive controls varied, with some studies using standard comparators such as alendronate sodium, BMP2, or Wnt3a, whereas others relied on vehicle- or untreated-groups [31, 32]. Specificity of Wnt signaling involvement was commonly verified using inhibitors including XAV939, IWP-2, Box5, LDN-193189, Dkk1, and Noggin (Lou et al., 2018; Wang et al., 2018; Liu et al., 2022; Luo et al., 2023; Xing et al., 2025). Despite this heterogeneity, the collective evidence consistently supports Wnt/β-catenin signaling as a convergent mediator of osteoanabolic responses. Importantly, these interventions affected not only osteoblast differentiation but also osteoclastogenesis and OPG/RANKL signaling, thereby reinforcing Wnt’s central role in bone remodeling balance.

### Effects on osteoblast differentiation and mineralization

Several classes of interventions enhanced osteoblast lineage commitment, extracellular matrix deposition, and mineralization through activation of Wnt/β-catenin signaling. Physical modulation using large-gradient high magnetic fields produced polarity-dependent effects in osteocyte macrophage-derived cell lines: upward fields suppressed viability and downregulated and Wnt proteins (Wnt1, β-catenin, LRP6) while increasing sclerostin (SOST), whereas downward fields enhanced proliferation, nitric oxide release, OPG expression, and Wnt-driven osteogenesis (Zhang et al., 2023).

Nanomaterials also demonstrated anabolic activity. In MC3T3-E1 and BMSC cultures, cerium oxide nanoparticles (CeO_2_NPs) promoted mineralization via upregulation of family with sequence similarity 53 (Fam53B) and nuclear β-catenin translocation, effects blocked by the Wnt inhibitor KYA1797K (Luo et al., 2023). Similarly, 6% strontium-doped mesoporous silica nanoparticles (6% Sr-MSNs) upregulated osteogenic markers (Runt-related transcription factor 2 (Runx2), BMP2, collagen type I (Col-1), osteopontin (OPN), osteocalcin (OCN)), suppressed GSK-3β, and activated β-catenin/c-Myc signaling. *In vivo*, these effects translated into enhanced bone mass and angiogenesis (Liu et al., 2022).

Biological derivatives also acted via Wnt signaling. Platelet-rich plasma (PRP) increased β-catenin and cyclin D1 expression and improved trabecular microarchitecture; inhibitor assays additionally revealed partial involvement of nuclear factor kappa-light-chain-enhancer of activated B cells/p38 (NF-κB/p38) signaling (Wang et al., 2018). Calcium hydroxide increased alkaline phosphatase (ALP) and OPN expression, although this effect appeared independent of Wnt activation (Cui et al., 2017).

Phytochemicals such as protosappanin B and schisandrin A reversed glucocorticoid-induced suppression of osteoblast differentiation by restoring ALP, Runx2, Osterix, OCN, and calcium deposition. These effects were abolished by Wnt inhibitors, confirming dependence on Wnt/β-catenin activity (Ai et al., 2025; Fan et al., 2025). Irisin treatment enhanced osteoblast activity and trabecular bone quality in aged mice, again via β-catenin activation (Xing et al., 2025). Likewise, (R)-dehydroxyabscisic alcohol β-D-apiofuranosy l-(1ˮ→6ʼ) β-D-glucopyranoside (DAG) promoted mineralization through coordinated BMP/Wnt activation, and inhibitory assays with Noggin or Dkk1 confirmed Wnt involvement (Liu et al., 2019).

Taurine exerted cytoprotective effects against oxidative stress by upregulating β-catenin, extracellular signal-regulated kinase (ERK), and nuclear factor erythroid 2–related factor 2 (Nrf2)-dependent antioxidant enzymes (heme oxygenase-1(HO-1), NAD(P)H quinone dehydrogenase 1 (NQO1), and glutamate–cysteine ligase catalytic subunit (GCLC)). DKK1 blockade abolished taurine’s protective activity, linking redox balance with Wnt activation in osteoblast survival (Lou et al., 2018).

Small molecules and synthetic modulators further supported these findings. H19, 5-(3-(4-(dimethylamino) phenyl) allylidene)-1-(3,5-dimethyl-phenyl) pyrimidine-2,4,6 (1H, 3H, 5H)-trione (DMP-PYT) was identified as a novel estrogenic, cell-permeable compound activating both BMP and Wnt pathways. *In vitro*, it increased ALP, Runx2, Osterix, OCN, and β-catenin–TCF/LEF activity, with synergy when combined with BMP2. In zebrafish, it enhanced skeletal formation, with BMP2 and Wnt3a serving as positive controls (Bae et al., 2017).

### Effects on osteoclastogenesis and bone remodeling balance

A subset of studies highlighted that Wnt modulation also impacted osteoclast differentiation and coupling signals. Magnetic field exposure significantly influenced osteoclastogenesis: upward fields promoted resorption through SOST upregulation and Wnt suppression, while downward fields reduced osteoclast differentiation (Zhang et al., 2023). PRP treatment inhibited osteoclastogenesis by decreasing RANKL-induced differentiation genes (tartrate-resistant acid phosphatase (TRAP), nuclear factor of activated T-cells, cytoplasmic 1 (NFATc1), and part of AP-1 transcription factor complex (c-Fos)), while simultaneously enhancing Wnt-mediated osteoblast activity (Wang et al., 2018). Similarly, 6% Sr-MSNs shifted the OPG/RANKL ratio favorably, reducing osteoclast activity while promoting coupled osteoblastogenesis and angiogenesis (Liu et al., 2022).

### Endogenous modulators of wnt signaling in bone remodeling

Endogenous regulators, including lncRNAs, transcription factors, RNA-binding proteins, and epigenetic modifiers, critically shape bone remodeling by modulating Wnt/β-catenin signaling. Studies were performed using *in vitro* models (hBMSCs, SCAPs, MC3T3-E1, preosteoblasts, osteoclast precursors) and *in vivo* models (knockout, and transgenic mice, including OVX-induced and aging-related osteoporosis). Unlike exogenous studies, most relied on genetic manipulation rather than pharmacological interventions. Lithium chloride was occasionally used as a Wnt activator to study Paired-like homeodomain transcription factor 1 (Pitx1) and calcium voltage-gated channel subunit alpha1 C, gene: CACNA1C (Cav1.2) rescue models effect on the Wnt pathway, but no standard anti-osteoporotic drugs were included (Fei et al., 2019; Karam et al., 2019). Overall, dysregulation of these intrinsic regulators contributes to senile and secondary osteoporosis, whereas restoring Wnt activity can rescue bone formation and prevent resorption.

### Effects on osteoblast differentiation and mineralization

Several regulatory axes were found to converge on the Wnt pathway. Silencing of Long Intergenic Non-Protein Coding RNA 1119 (LINC01119) enhanced osteogenic differentiation of hBMSCs by increasing Wnt receptor expression (LRP5, β-catenin), an effect abrogated by co-silencing FZD4 (Gao et al., 2022). The RNA-binding protein Quaking (QKI) acted as a negative regulator by binding and destabilizing transcripts of dishevelled 3 (Dvl3), Fzd7, and β-catenin; its knockdown restored osteogenesis both *in vitro* and *in vivo* (Yan et al., 2023). Transgenic overexpression of Pitx1 suppressed osteogenic markers (Runx2, ALP, osterix, OCN), and increased Wnt inhibitors (Sost, Dkk1), whereas lithium chloride treatment rescued Wnt activity and bone formation (Karam et al., 2019). Similarly, knockdown of Guanine nucleotide binding protein subunit α (GNAS) suppressed Wnt3a and β-catenin and impaired osteogenesis, whereas overexpression restored these parameters (Zheng et al., 2020).

Epigenetic mechanisms also emerged. In ovariectomized mice, reduced expression of General Control Non-derepressible 5 (GCN5) correlated with loss of β-catenin signaling and histone Histone H3 lysine 9 acetylation (H3K9) acetylation at Wnt gene promoters, impairing osteogenesis. GCN5 overexpression reversed this phenotype (Jing et al., 2018). In aging mice, Cav1.2 deficiency suppressed Runx2, ALP, OCN, nuclear β-catenin, and downstream cyclin D1/c-myc expression; restoration of Cav1.2 or pharmacological activation with Bay K8644 re-engaged Wnt signaling and osteogenic outcomes (Fei et al., 2019).

Additional regulatory interactions were identified. In SCAPs, elevated non-canonical Wnt5a suppressed osteogenic differentiation despite β-catenin activation, establishing a negative feedback loop via TAZ downregulation (Fu et al., 2022). In preosteoblast cultures and mouse models, Notch activation antagonized Wnt signaling during osteoblast-to-osteocyte transition; pharmacological Notch inhibition with N-[N-(3,5-Difluorophenacetyl)-L-alanyl]-S-phenylglycine t-butyl ester (DAPT) restored β-catenin and protein kinase B (AKT) activity, partially normalizing remodeling (Shao et al., 2018).

### Effects on osteoclastogenesis and bone remodeling

Pitx1 exerts effects beyond osteoblastogenesis. Pitx1 overexpression enhanced osteoclast number, RANKL/OPG ratio, and resorptive activity, aggravating osteoporotic bone loss through inhibiting the Wnt signalling pathway (Karam et al., 2019).

### Clinical and epidemiological evidence supporting wnt pathway involvement in osteoporosis

Findings from databases analysis, biomarker-based cohort studies, and genetic association research all point to a consistent link between Wnt pathway dysregulation and osteoporosis, even though the study designs and populations differed. Since these were observational and correlative studies, positive controls were not applicable. However, across studies, reduced β-catenin activity and elevated levels of Wnt inhibitors such as DKK1 were repeatedly associated with higher osteoporosis risk and disease progression.

In the epidemiological study, databases analysis of postmenopausal Caucasian women identified 12 Wnt-related DEGs. Machine learning models (SVM, RF, GLM, XGB) demonstrated predictive value, suggesting Wnt-related gene expression patterns are early indicators of low BMD, as RAC1, Kelch-like protein 12 (KLHL12), and GSK3B are increased in low BMD and 7 others are decreased in low BMD. Further analysis identified several molecular compounds and drugs that may modulate the Wnt pathway: sotrastaurin, β-sitosterol, palbinone, and fluoxetine were linked to GSK3B regulation, while vemurafenib and dabrafenib were associated with Ras-related C3 botulinum toxin substrate 1 (RAC1) expression. These findings highlight the involvement of the Wnt pathway in osteoporosis (Lai et al., 2024).

In the clinical cohort study on 120 patients, vertebral compression fracture cases showed a decrease in β-catenin, interleukin-10 (IL-10), transforming growth factor beta 1 (TGF-β1), and bone formation markers, but DKK1, pro-inflammatory cytokines, and resorption markers were increased, confirming Wnt suppression drives bone fragility (Ma et al., 2021).

In the genetic study on 1,198 Chinese Han individuals, LRP5 rs11228240 reduced osteoporosis risk, while AXIN1 variants (rs9921222, rs2301522) increased susceptibility, underscoring genetic modulation of Wnt signaling in osteoporosis (Cui et al., 2022).

## Discussion

The canonical Wnt/β-catenin signaling pathway is central to skeletal biology, regulating osteoblast differentiation, bone matrix formation, and the suppression of osteoclast activity (Janssen et al., 2021). This review synthesizes current evidence on endogenous and exogenous modulators of the pathway, as well as clinical implications, highlighting its therapeutic potential for osteoporosis. Several modulators show promising clinical potential in osteoporosis management through stimulation of the Wnt signaling pathway. Agents such as CeO_2_NPs, DAG, taurine, irisin, protosappanin B, schisandrin A, and DMP-PYT have been reported to stimulate osteogenesis, supporting bone formation (Bae et al., 2017; Lou et al., 2018; Liu et al., 2019; Luo et al., 2023; Ai et al., 2025; Fan et al., 2025; Xing et al., 2025). Meanwhile, interventions like 12 T LG-HMF (magnetic stimulation), 6% Sr-MSNs, and platelet-rich plasma (PRP) not only promote osteoblast activity but also inhibit osteoclastogenesis (Wang et al., 2018; Liu et al., 2022; Zhang et al., 2023), thereby reducing bone resorption. Collectively, these modulators demonstrate how targeting the Wnt pathway can influence both bone formation and resorption, highlighting their translational potential in osteoporosis therapy.

### The wnt pathway crosstalk with other pathways

Bone remodeling rarely depends on a single pathway. Synergistic activation of Wnt and BMP pathways by 6% Sr-MSNs or DMP-PYT underscores the integrative nature of osteogenesis (Bae et al., 2017; Liu et al., 2022). In contrast, Notch signaling often counterbalances Wnt activity: activation of Notch reduces β-catenin and osteoblast proliferation, while its inhibition restores Wnt signaling (Shao et al., 2018). Non-canonical Wnt pathways, such as Wnt5a-mediated signaling, introduce an additional layer of complexity in bone biology. Interestingly, the effects of Wnt5a are cell-type dependent. In stem cells from the apical papilla (SCAPs), Wnt5a suppresses the canonical Wnt/β-catenin pathway, thereby inhibiting osteoblast differentiation. In contrast, in the MC3T3-E1 pre-osteoblast cell line, Wnt5a stimulation enhances osteoblast activity and promotes differentiation. This dual behavior suggests that Wnt5a does not act in a uniform manner but instead exerts context-dependent effects, likely influenced by the cellular environment, receptor availability. Oxidative stress responses are also closely linked to Wnt activity (Fu et al., 2022). Agents such as taurine restored osteoblast viability under oxidative stress by activating Wnt alongside ERK/Nrf2 antioxidant pathways, suggesting a protective role for Wnt in hostile microenvironments (Lou et al., 2018).

### Systemic implications

Despite promising experimental outcomes, Wnt-targeted therapy must be approached with caution. Wnt signaling is conserved across multiple tissues, influencing proliferation, differentiation, and homeostasis beyond bone (Strubberg et al., 2018; Lin et al., 2023; Yalcin et al., 2023). Aberrant activation is implicated in cancers such as colorectal, breast, and liver carcinomas, making systemic modulation a double-edged sword (Tang et al., 2019; Yu et al., 2021; Xu et al., 2022; He and Gan, 2023). For instance, porcupine o-acyltransferase (PORCN) inhibitors under development for oncology reduce tumor growth but also impair bone integrity, lowering bone mineral density and increasing fracture risk through Wnt pathway inhibition (Xi and Chen, 2014). This raises concerns for patients on long-term anti-Wnt therapies, especially those with pre-existing skeletal fragility.

Bone metabolism is tightly regulated by endogenous modulators of the Wnt pathway, and altering their expression carries both therapeutic potential and biological risks. For instance, LINC01119 knockdown enhanced osteogenesis, whereas FZD4 knockdown abolished this effect, highlighting the fragility of the LINC01119–FZD4–Wnt axis (Gao et al., 2022). However, LINC01119 knockdown modulates adipogenesis in mesenchymal stem cells (Gao et al., 2022), controls pro-tumorigenic activity associated with the suppressor of cytokine signaling 5 (SOCS5) protein (Tu et al., 2021), and contributes to neuropathic pain by potentially regulating pain-related pathways in nerve injuries (Zhang et al., 2021). Similarly, QKI suppression promoted osteogenic markers by stabilizing Dvl3, Fzd7, and β-catenin, but its overexpression suppressed bone formation, suggesting its dual regulatory nature (Yan et al., 2023). However, QKI suppresses the growth and malignant transformation of lung cancer cells in both *in vitro* and *in vivo* models (Zong et al., 2014; Cao et al., 2021). In contrast, Pitx1 overexpression induced osteoporosis by repressing Runx2, ALP, osterix, and OCN while increasing Wnt inhibitors (Sost, Dkk1); lithium chloride partly reversed this, showing how Wnt reactivation may counter senescence-driven bone loss (Karam et al., 2019). Also, PITX1 interacts with p53 and promotes tumor progression and metastasis in esophageal, gastric, colorectal, and liver cancers (Zhao and Xu, 2023). Negative regulators were also evident in the Wnt5a/β-catenin/TAZ loop, where Wnt5a suppressed osteogenesis and blocked TAZ rescue effects, underlining the risk of excessive Wnt5a activity (Fu et al., 2022). However, Wnt5a acts as an antitumor factor in leukemia, counteracting multiple oncogenic processes associated with the disease (Bueno et al., 2023). Moreover, GNAS knockdown impaired ALP, Runx2, and Wnt3a–β-catenin signaling, whereas overexpression restored osteogenesis (Zheng et al., 2020). However, GNAS knockdown can cause G protein subunit alpha-s (Gsα) deficiency, which is implicated in several conditions, including certain types of pseudohypoparathyroidism that are often characterized by severe obesity beginning in early childhood (Abbas et al., 2024). In contrast, epigenetic regulation was seen with GCN5, where its loss reduced β-catenin activation at Wnt promoters, while overexpression restored bone mass (Jing et al., 2018). However, inhibiting GCN5 could be a positive therapeutic strategy for treating acute lymphoblastic leukemia by reducing the levels of the E2A-PBX1 oncoprotein (Holmlund et al., 2013). Likewise, Zmpste24 deficiency impaired Runx2, ALP, OCN, and Wnt target genes, but Cav1.2 activation rescued these defects (Fei et al., 2019). Also, Cav1.2 channel plays an important role in calcium influx regulating in cardiomyocytes, which is essential for excitation–contraction coupling and maintaining normal heart rhythm and contractility (Westhoff and Dixon, 2021).

### Clinical translational perspectives

Reviewed studies have highlighted several gene regulators whose presence has been linked to osteoporosis and that may also hold potential clinical significance (Karam et al., 2019; Gao et al., 2022; Yan et al., 2023). For instance, QKI might be suggested as a biomarker for glucocorticoid-induced osteoporosis, LINC01119 might be identified as a marker associated with osteoporosis risk, and PITX1 might be linked to senile osteoporosis. While their translation into routine clinical practice remains at an early stage, these findings provide important insights into patient-specific disease mechanisms and may open future opportunities for precision diagnostics and targeted therapies.

Moreover, the epidemiological study is powerful because it uses large datasets to pinpoint specific Wnt-related genes (like RAC1, GSK3B) that are strongly linked to low bone mineral density (BMD) (Lai et al., 2024). This gives us a solid list of “suspects” to investigate. The fact that diagnostic models (like SVM and RF) could be built using these genes suggests they have real clinical relevance for identifying at-risk individuals. Crucially, it also connected these genes to existing molecular compounds—like vemurafenib for RAC1 and sotrastaurin or fluoxetine for GSK3B—providing an immediate link to potential therapeutic agents that could modulate this pathway for bone health (Lai et al., 2024). The clinical study on fracture patients extends the findings by demonstrating their relevance in patients, directly linking dysregulation of the Wnt pathway—characterized by reduced β-catenin and elevated DKK1—to impaired bone remodeling. The clear biological story was well established: the disrupted Wnt signaling leads to increased bone breakdown (high RANKL and Beta-C-terminal telopeptide of type I collagen (β-CTX)) and decreased bone formation (low OPG and Procollagen type I N-terminal propeptide (PINP)) (Ma et al., 2021). This provides a mechanistic explanation for why targeting this pathway could treat the disease, not just diagnose it. Finally, the genetics study adds another critical layer. It shows that natural variations in key Wnt pathway genes (LRP5 and AXIN1) directly influence a person’s risk of developing osteoporosis (Cui et al., 2022). The fact that a specific variant in LRP5 can cut the risk in half is particularly compelling evidence that these are not just biomarkers, but potential levers we could pull with a drug. So, to clarify: the combined results suggest that these genes (especially like LRP5 and AXIN1) are not just passive indicators. They appear to be active players in causing or preventing the disease. Therefore, developing drugs that inhibit negative regulators (like AXIN1) or promote positive regulators (like LRP5) could be a valid treatment strategy—including those computationally identified—could effectively treat bone metabolic diseases. The next essential step is clinical research into these potential therapies.

### Limitations and outlook

Current findings highlight the potential of Wnt pathway modulation for osteoporosis, but several gaps remain. Most studies rely on preclinical models, often without pharmacological positive controls and using different models, making translational interpretation difficult. Moreover, while regulators such as LINC01119, PITX1, and QKI clearly modulate Wnt signaling *in vitro* or in mice, their relevance and side effects in human bone disease is not well established. Focusing solely on the Wnt pathway also risks oversimplification, as bone metabolism is shaped by a network of interconnected pathways. Another limitation relates to the time frame of our review: although we initially captured studies published between June 2017 and February 2024, we have since updated our search to include literature up to August 2025. Despite this, some relevant studies may still not have been captured, and the complexity of the Wnt pathway cannot be fully addressed in a single scoping review. Future research should integrate multi-pathway analysis and prioritize clinical validation to bridge preclinical discoveries with patient care.

**FIGURE 2:**
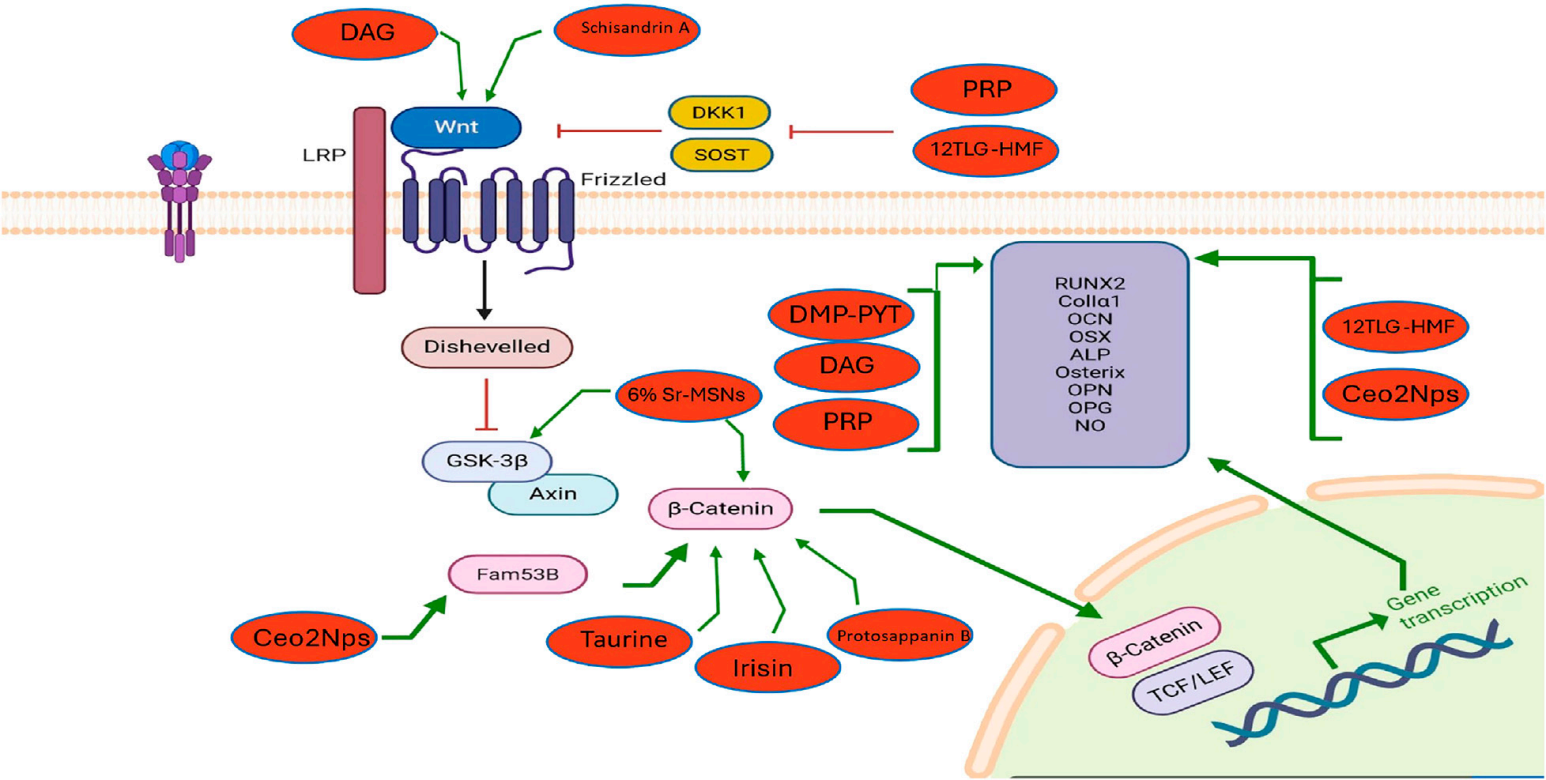
The influence of exogenous modulators on the Wnt/β-catenin signaling pathway in osteoblasts and their effects on osteogenesis and osteoporosis. The diagram illustrates how external agents such as 12TLG-HMF, PRP, DAG, DMP-PYT, CeO_2_NPs, Taurine, Irisin, Protosappanin B, Schisandrin A, and 6% Sr-MSNs activate the Wnt/β-catenin pathway in osteoblasts. These agents enhance β-catenin stabilization, leading to nuclear translocation and transcription of osteogenic genes (RUNX2, ALP, OPG, NO, etc.).

**FIGURE 3:**
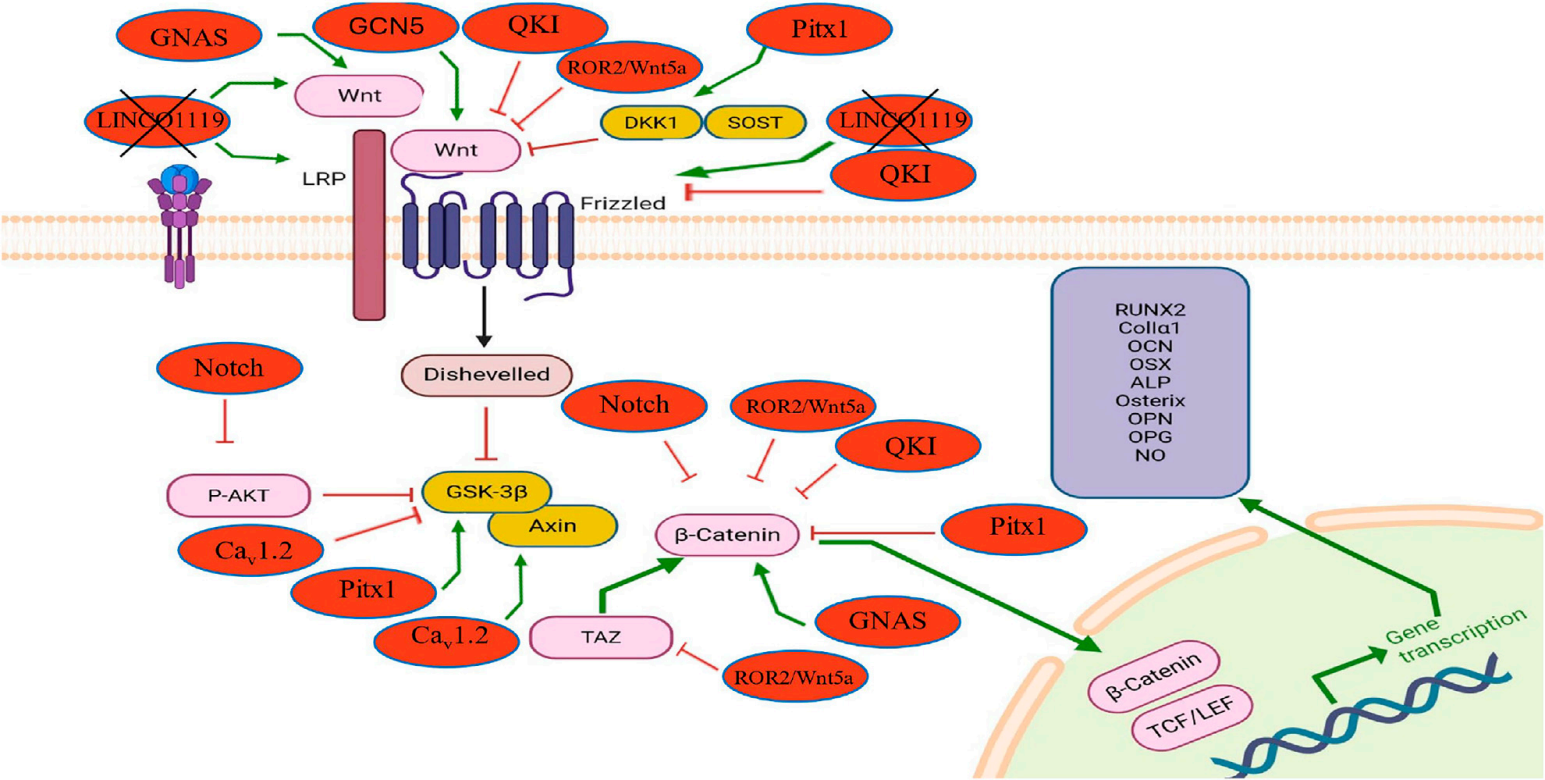
The influence of endogenous modulators on the Wnt/β-catenin signaling pathway in osteoblasts and their effects on osteogenesis and osteoporosis. This diagram shows how internal regulators such as GNAS, Caᵥ1.2, and TAZ activate the Wnt/β-catenin pathway, enhancing osteogenic gene expression (RUNX2, ALP, OPG, etc.). In contrast, molecules like QKI, Pitx1, Notch, and LINC01119 inhibit Wnt signaling by suppressing β-catenin activity or upstream components. The figure also highlights pathway crosstalk of non-canonical Wnt modulators (ROR2/Wnt5a) that influence osteoblast differentiation and bone remodeling through the canonical Wnt.

Table 2 and Figures 2, 3 below concluded that stimulators of the Wnt pathway improve osteoporosis by promoting osteogenesis and suppressing osteoclastogenesis. In contrast, inhibitors of the Wnt pathway contribute to osteoporosis by reducing osteogenic activity and enhancing osteoclastogenesis. Collectively, these consistent findings demonstrate that activation of the Wnt pathway enhances bone remodeling, whereas its inhibition leads to decreased bone remodeling.

**TABLE 2:** Summary of exogenous and endogenous modulators modulating the Wnt pathway in relation to osteoporosis.

Modulators	Type/Class	Effect on Wnt pathway	Effect on osteoporosis
Exogenous modulators			
12 T LG-HMF downward	Physical factor	Stimulates	Improves osteoporosis
Cerium oxide nanoparticles (Ceo_2_Np_s_)	Nanoparticle	Stimulates	Improves osteoporosis
6% Sr-MSNs	Nanoparticle	Stimulates	Improves osteoporosis
Platelet-rich plasma (PRP)	Biological product	Stimulates	Improves osteoporosis
Calcium hydroxide	Inorganic compound	Non	Improves osteoporosis
taurine	Amino acid	stimulates	Improves osteoporosis
DAG	Phytochemical	stimulates	Improves osteoporosis
Irisin	Phytochemical	stimulates	Improves osteoporosis (Age-related osteoporosis)
Protosappanin B	Phytochemical	stimulates	Improves osteoporosis (Glucocorticoid-induced osteoporosis)
Schisandrin A	Phytochemical	stimulates	Improves osteoporosis (Glucocorticoid-induced osteoporosis)
DMP-PYT	Synthetic compound	stimulates	Improves osteoporosis
Endogenous modulators			
LINC01119	lncRNA	Inhibits	Induces osteoporosis
Wnt5a	Protein	Inhibits	Induces osteoporosis
QKI	RNA-binding protein	Inhibits	Induces osteoporosis (Glucocorticoid-induced osteoporosis)
Pitx1	Transcription factor	Inhibits	Induces osteoporosis (Senile osteoporosis)
GNAS	Protein	Stimulates	Improves osteoporosis
GCN5	Enzyme	Stimulates	Improves osteoporosis (Postmenopausal osteoporosis)
Ca_v_1.2	Ion channel	Stimulates	Improves osteoporosis (Age‐related osteoporosis)
Notch pathway	Signaling pathway	Inhibits	Induces osteoporosis

## Conclusion

This review underscores the pivotal role of the canonical Wnt/β-catenin pathway in regulating bone remodeling and its therapeutic relevance in osteoporosis. Various endogenous and exogenous modulators influence Wnt activity to promote osteogenesis and inhibit osteoclastogenesis. While Wnt-targeted therapies hold promise, systemic modulation carries risks due to the pathway’s roles in other tissues and in cancer biology. Therefore, selective and localized approaches are essential to avoid off-target effects. Future research should emphasize balanced remodeling models, long-term safety, and precision-targeted delivery to fully harness the therapeutic potential of Wnt signaling in osteoporosis.
